# Creating a sustainable brain health navigator model to improve the diagnostic journey for Alzheimer's disease: the experience of one health care system in New Hampshire

**DOI:** 10.1002/alz70860_101261

**Published:** 2025-12-23

**Authors:** Karen Blackmon, Dax C. Volle, Corie C. Crane

**Affiliations:** ^1^ Dartmouth Hitchcock Medical Center | Psychiatry Department, Lebanon, NH, USA; ^2^ Geisel School of Medicine at Dartmouth, Hanover, NH, USA

## Abstract

**Background:**

Dartmouth Health (DH) is an academic medical center and health system, as well as the largest healthcare organization in New Hampshire. Our community hospitals and clinics serve patients across New Hampshire, Vermont, and Maine. Our outreach in rural underserved populations includes a long history of implementing integrative and collaborative care models into primary care. However, a major gap remains in early detection of Alzheimer's Disease and Related Dementias (ADRD). There is interest at the leadership level in addressing this gap by improving and harmonizing early detection of ADRD across the numerous primary care settings within DH. The Brain Health Navigator (BHN) Program will help to fill this gap using evidenced‐based guidelines and iterative learnings from Community of Practice sessions, in collaboration with other health systems.

**Methods:**

Initial implementation of the BHN pilot will bridge primary care (Departments of General Internal Medicine and Community and Family Medicine) with specialty care (Departments of Psychiatry and Neurology) at DH Medical Center. Quality improvement metrics such as care flow efficiency, patient experience, and provider productivity will be tracked for the 6‐month duration of the program to determine the added value of the BHN program. These metrics will inform ongoing efforts among leadership to harmonize early ADRD detection across the DH system. Electronic health records will be optimized for decision support, workflows, built‐in alerts for decline in cognitive screen scores, and provision of educational materials. Regular communication between DH BHN champions and DH leadership has the potential to integrate effective care innovations at the system‐wide level, which will support the sustainability of early detection programs.

**Results:**

DH envisions a future clinical practice for detecting cognitive impairment at the primary care level using a toolkit that combines blood‐based biomarkers and digital cognitive screening tools. Such tools will require minimal clinician time to administer and interpret. Brain health navigators will guide screen‐positive patients through the flow of follow‐up consultations to avoid dropped care.

**Conclusions:**

The BHN model will support the implementation of boundary spanning roles across primary and specialty care, designed for a timely and accurate diagnostic journey at DH, that may be applicable to other large rural health systems.

